# Supplementary figures and images for: CTDSP1 inhibitor rabeprazole regulates DNA-PKcs dependent topoisomerase I degradation and irinotecan drug resistance in colorectal cancer
Source: PLoS One. 2020 Aug 7;15(8):e0228002. doi: 10.1371/journal.pone.0228002 (PMC7413750; doi:10.1371/journal.pone.0228002)

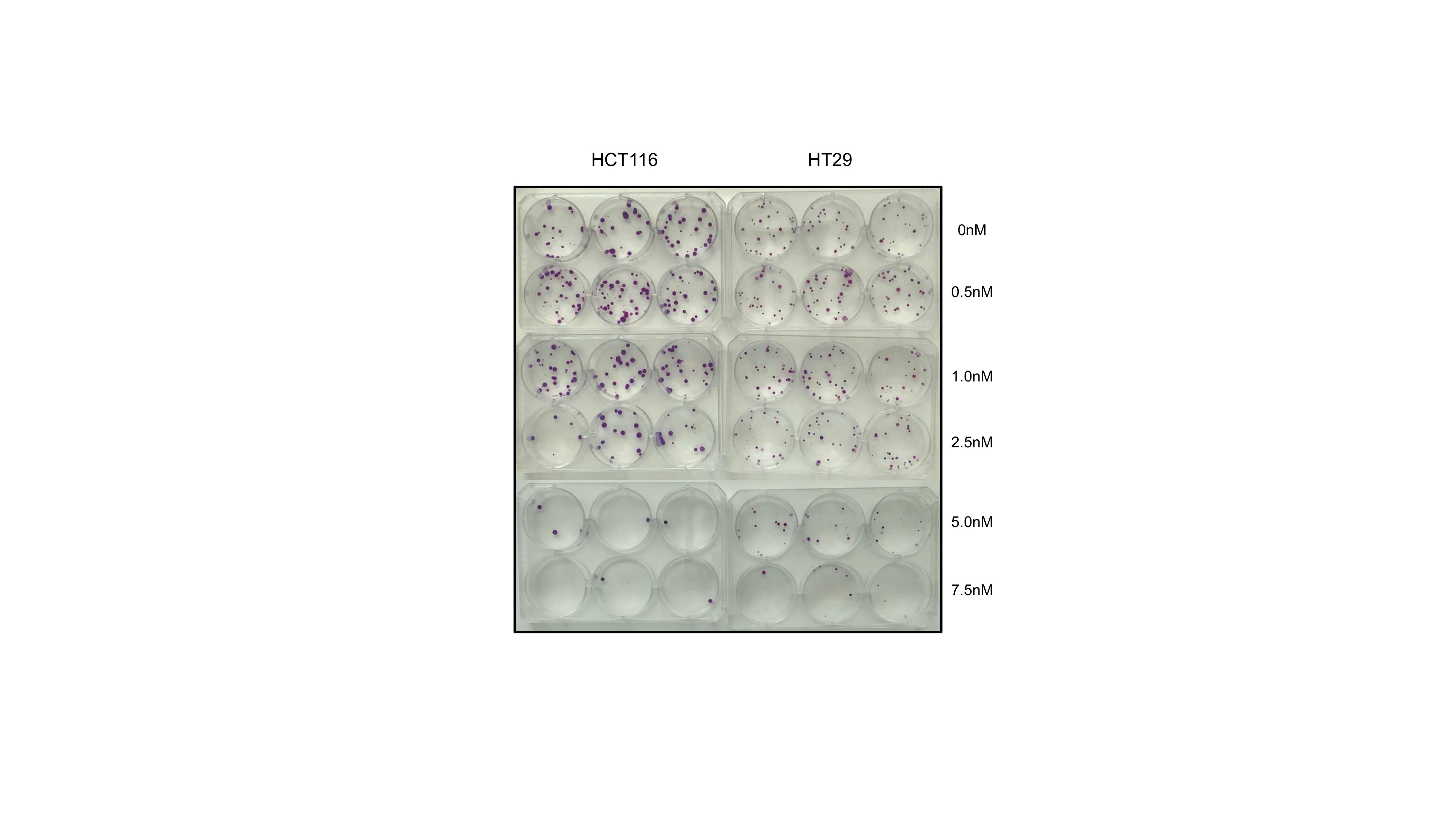

Supplement: S1 Fig — HCT116 and HT29 cells were treated with various concentrations of SN-38 and clonogenic assays were performed to determine the relative number of colonies. (TIF) [file pone.0228002.s001.tif]

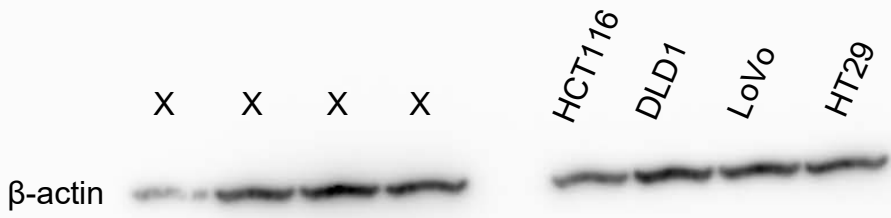

Supplement: S1 Data — (ZIP) [file pone.0228002.s003.zip › PLOS ONE WB data/Figure 1/Fig1A actin raw images.pdf]

CTDSP1

HCT116

DLD1

LoVo

HT29

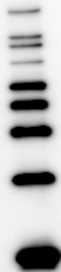

Supplement: S1 Data — (ZIP) [file pone.0228002.s003.zip › PLOS ONE WB data/Figure 1/Fig1A CTDSP1 raw images.pdf]

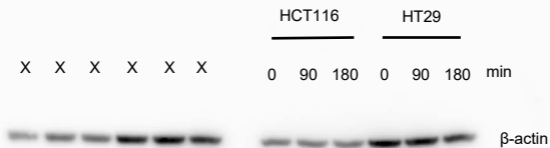

Supplement: S1 Data — (ZIP) [file pone.0228002.s003.zip › PLOS ONE WB data/Figure 1/Fig1B actin raw images.pdf]

X X X X X X

HCT116

HT29

0 90 180 0 90 180 min

Topol

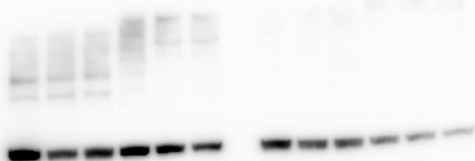

Supplement: S1 Data — (ZIP) [file pone.0228002.s003.zip › PLOS ONE WB data/Figure 1/Fig1B Topo1 raw images.pdf]

X

X

siCTDSP1 control

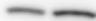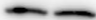

$\beta$ -actin

Supplement: S1 Data — (ZIP) [file pone.0228002.s003.zip › PLOS ONE WB data/Figure 2/Fig2A actin raw images.pdf]

X

X

siCTDSP1

control

CTDSP1

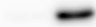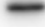

Supplement: S1 Data — (ZIP) [file pone.0228002.s003.zip › PLOS ONE WB data/Figure 2/Fig2A CTDSP1 raw images.pdf]

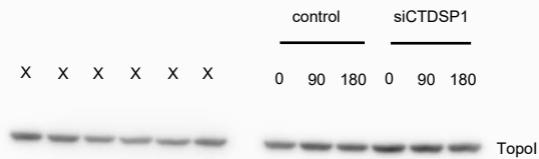

Supplement: S1 Data — (ZIP) [file pone.0228002.s003.zip › PLOS ONE WB data/Figure 2/Fig2B actin raw images.pdf]

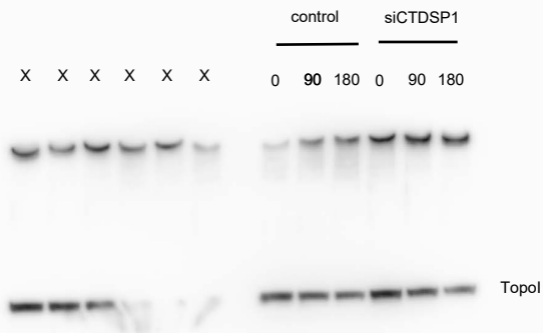

Supplement: S1 Data — (ZIP) [file pone.0228002.s003.zip › PLOS ONE WB data/Figure 2/Fig2B Topo1 raw images.pdf]

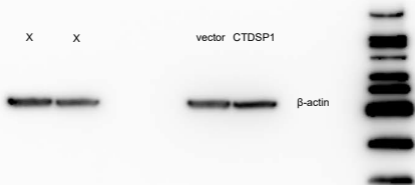

Supplement: S1 Data — (ZIP) [file pone.0228002.s003.zip › PLOS ONE WB data/Figure 3/Fig3A actin raw images.pdf]

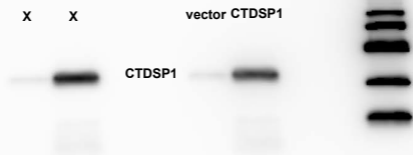

Supplement: S1 Data — (ZIP) [file pone.0228002.s003.zip › PLOS ONE WB data/Figure 3/Fig3A CTDSP1 raw images.pdf]

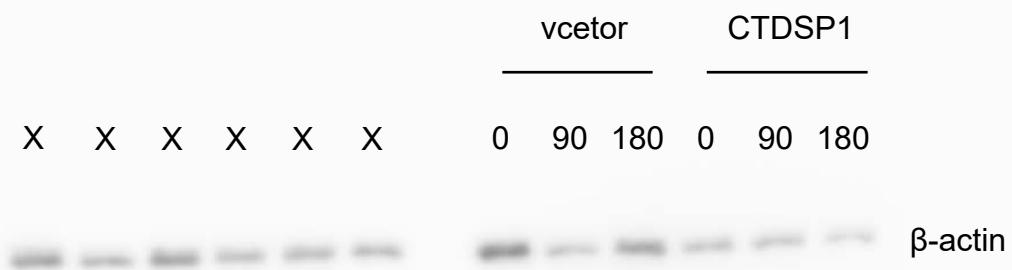

Supplement: S1 Data — (ZIP) [file pone.0228002.s003.zip › PLOS ONE WB data/Figure 3/Fig3B actin raw images.pdf]

X X X X X X

vector

CTDSP1

0 90 180

0 90 180

Topol

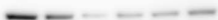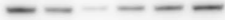

Supplement: S1 Data — (ZIP) [file pone.0228002.s003.zip › PLOS ONE WB data/Figure 3/Fig3B Topo1 raw images.pdf]

X

X

HCT116 HT29

GAPDH

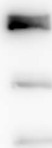

Supplement: S1 Data — (ZIP) [file pone.0228002.s003.zip › PLOS ONE WB data/Figure 4/Fig4A GAPDH raw images.pdf]

X

X

HCT116 HT29

pDNAPKcs

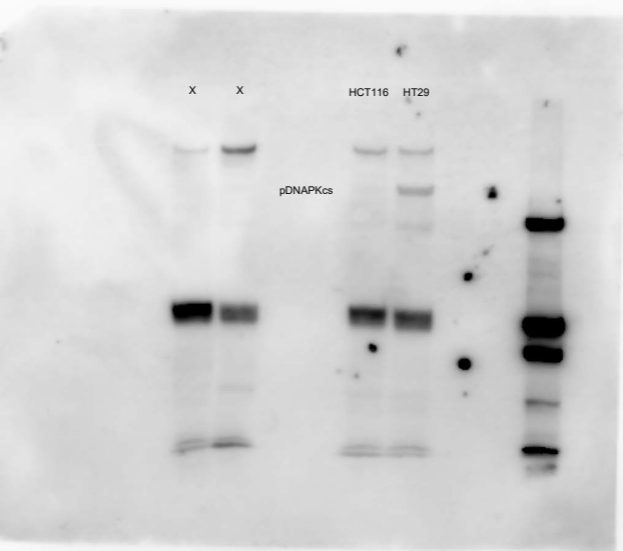

Supplement: S1 Data — (ZIP) [file pone.0228002.s003.zip › PLOS ONE WB data/Figure 4/Fig4A pDNAPKcs raw images.pdf]

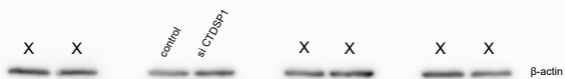

Supplement: S1 Data — (ZIP) [file pone.0228002.s003.zip › PLOS ONE WB data/Figure 4/Fig4B actin raw images.pdf]

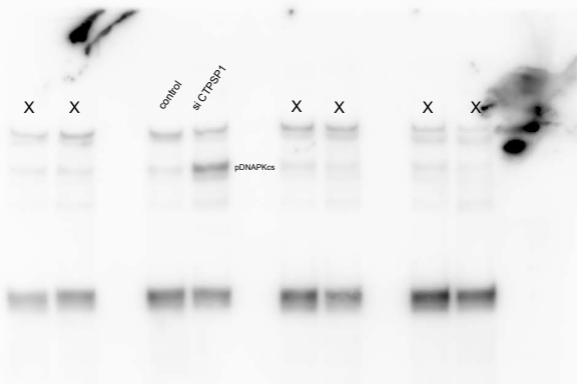

Supplement: S1 Data — (ZIP) [file pone.0228002.s003.zip › PLOS ONE WB data/Figure 4/Fig4B pDNAPKcs raw images.pdf]

control

si CTDSP1

—

+

—

+

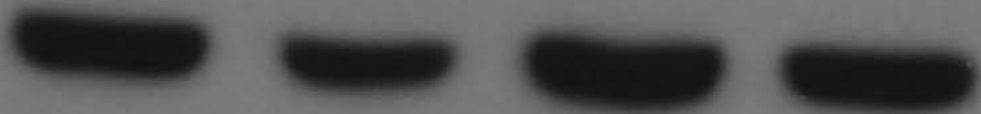

Supplement: S1 Data — (ZIP) [file pone.0228002.s003.zip › PLOS ONE WB data/Figure 4/Fig4C actin raw images.pdf]

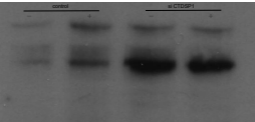

Supplement: S1 Data — (ZIP) [file pone.0228002.s003.zip › PLOS ONE WB data/Figure 4/Fig4C pDNAPKcs raw images.pdf]

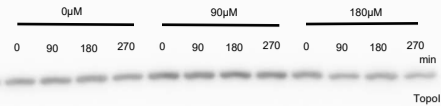

Supplement: S1 Data — (ZIP) [file pone.0228002.s003.zip › PLOS ONE WB data/Figure 5/Fig5A actin raw images.pdf]

0μM                      10μM                      20μM

0    90    180    270    0    90    180    270    0    90    180    270    min

Topol

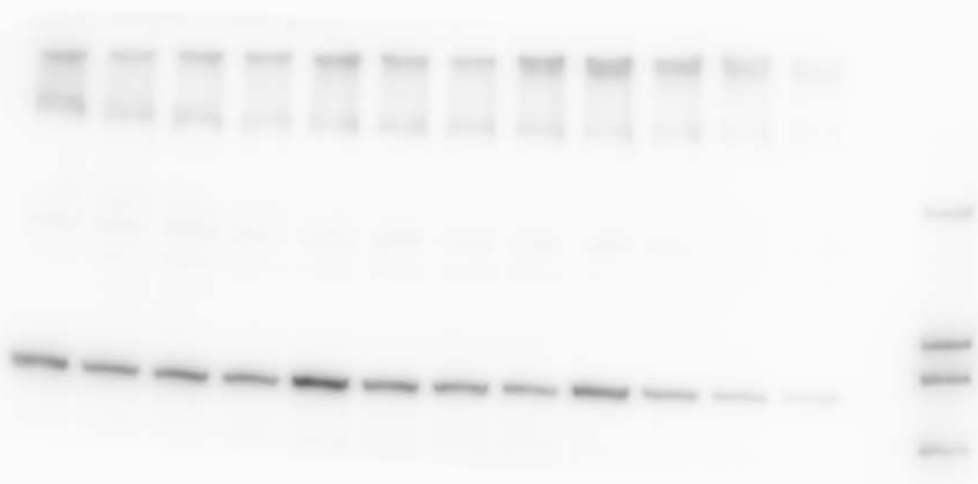

Supplement: S1 Data — (ZIP) [file pone.0228002.s003.zip › PLOS ONE WB data/Figure 5/Fig5A Topo1 raw images.pdf]

Rabeprazole

(-)

(+)

0

90

180

0

90

180

$\beta$ -actin

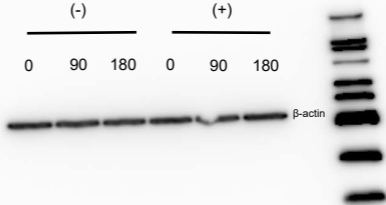

Supplement: S1 Data — (ZIP) [file pone.0228002.s003.zip › PLOS ONE WB data/Figure 5/Fig5B actin raw images.pdf]

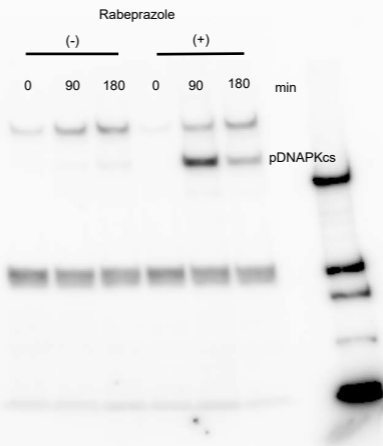

Supplement: S1 Data — (ZIP) [file pone.0228002.s003.zip › PLOS ONE WB data/Figure 5/Fig5B pDNAPKcs raw images.pdf]
