# Supplementary material for: CTDSP1 inhibitor rabeprazole regulates DNA-PKcs dependent topoisomerase I degradation and irinotecan drug resistance in colorectal cancer
Source: PLoS One. 2020 Aug 7;15(8):e0228002. doi: 10.1371/journal.pone.0228002 (PMC7413750; doi:10.1371/journal.pone.0228002)
Supplement: Attachment — Submitted filename: renamed_f0116.doc [file pone.0228002.s004.doc]

March 25 2020

Joerg Heber

Editor-in-Chief

*PLOS ONE*

Dear Editor:

Thank you for inviting us to submit a revised draft of our manuscript entitled, “CTDSP1 inhibitor rabeprazole regulates DNA-PKcs dependent topoisomerase I degradation and irinotecan drug resistance in colorectal cancer.” to PLOS ONE. We also appreciate the time and effort you and each of the reviewers have dedicated to providing insightful feedback on ways to strengthen our paper. Thus, it is with great pleasure that we resubmit our article for further consideration. We have incorporated changes that reflect the detailed suggestions you have graciously provided. We also hope that our edits and the responses we provide below satisfactorily address all the issues and concerns you and the reviewers have noted.

Blot/ gel image data are in supporting information.

To facilitate your review of our revisions, the following is a point-by-point response to the questions and comments delivered in your letter.

**Reviewer #1:**

Major comments:
1. In Figure 1, the authors should quantify the intensity of each blots compared to beta-actin protein levels. In Figure 1B, the authors should determine the phosphorylated Topo I levels.

Re: As suggested we have quantitatively analyzed the immunoblot intensity. The suggestion to include phospho-topoI immunoblot is very thoughtful. However, Our phospho-topoI-pS10 antibody is specific for immunohistochemistry and not for immunoblot analysis and there is no commercially available antibody.

2. The authors treated cells with 2.5 μM SN-38 in Figure 1B but treated cells with SN-38 at concentrations lower than 7.5 nM in Figure 1C. Similar views also found in Figures 2, 3 and 5. Is it a typo? Otherwise, it is hard to believe that TopoI protein levels determine the therapeutic effectiveness of SN-38. Unless the authors show that TopoI protein degradation is visible at the treatment with SN-38 at low nM range.

Re: The authors would humbly like to mention that depending on the type of assay the topoI drug concentration varies from micro-molar range to nano-molar. These assays were optimized decades ago by the leading scientists in the field and have been used since to understand the biology. Notable among the scientists who have established these assays are Dr. Leroy Liu and Dr. Yves Pommier. The references in this manuscript that support our statement are: #1, #12, #22, #23 and #24

3. In Figure 2B, the remarkable protein degradation should be detected in CTDSP1 knockdown cells at time 0 in comparison with control cells. However, it seems to be not. Please describe it.

Re: We would like to mention that in Fig. 3B, we have not deleted but overexpressed CTDSP1 in HT29 cells. It is important to mention that CTDSP1 expression in HT29 is minimal and overexpression inhibits SN-38 (irinotecan active metabolite) induced topoI degradation. At time 0 there is similar topoI expression in vector alone or CTDSP1 expressing cells.

4. The experimental procedure to generate TopoI-EGFP fusion by CRISPR/Cas9 system is not clearly presented. Please provide the sequence of sgRNA used in this study and sequencing result for the gene fusion.

Re: As suggested, we have included the detailed experiments procedure of topoI-GFP fusion protein generation by CRISPR/CAS9. We have also provided all the relevant sequences, including sgRNA used in supplementary data.

5. What is the protein band for pDNA-PKcs in Figure 4B?

Re: DNA-PKcs full-length protein is approximately 450kDa. In Fig. 4B a cleaved fragment of the protein containing phosphorylated S2056 was identified at 250 kDa.

6. In Figure 5A, the protein levels of Topo 1 at time 0 seem to be not equal. Why?

Re: We agree that there is a minor difference in at 0 time point. But the rate of the change in rate of degradation due to rabeprazole is significantly higher.

7. In Table 1, if the clinical data from a small sample size is capable of supporting the conclusion that rabeprazole had an effect on the response to irinotecan in clinical settings?

Re: We conclude that rabeprazole has effect on rate of topoI degradation by multiple ways in cell based assay. The clinical data is supportive of these findings. The number of patients who had received rabeprazole and irinotecan is very small (n=6) and thus it cannot be conclusive. In the revised manuscript we have included a statement of this effect.

Minor comments:
1. The authors should describe statistical tools they used in MM section

Re: We have included the description of statistical tools in revised manuscript.

2. I found many typos and grammar errors in the manuscript. So, English editing of this manuscript should be needed.

Re: We have edited the revised manuscript.
 **Reviewer #2:**

1: The expressions of CTDSP1 and Topo I should be evaluated and quantified in western blot and Q-PCR analysis. This includes figure 1, 2, 3 and 5.

Re: As suggested, we have quantitatively analyzed the immunoblot in Fig. 1, 2, 3, and 5. As our work largely deals with proteasomal degradation, we understand that Q-PCR based any quantitation of mRNA will not provide any additional information on protein quantities or rate of protein degradtion.

2: The appropriate internal control of pDNAPKcs is total form of DNAPKcs. Please add this important data with individual pDNAPKcs western blot.

Re: DNA-PKcs is a 450 kDa protein and immunoblot analysis of the protein provides multiple bands. This is well documented in literature as well as in our study (Fig.4). These are both natural protease cleavage product during cell lysis and some naturally found cleaved fragments of the full-length protein. Thus it is very difficult to provide data of total full length of DNA-PKcs as control.

3: p-ATM, ATM, p-ATR, ATR, gimma H2AX and XRCC4 protein expressions are always changed, followed by the activation of pDNAPKcs. The authors should also evaluate these proteins.

Re: We agree with the suggestion of reviewer that protein expression determination of the stated protein will provide information on DNA-DSB response pathway however it will not provide any additional information on upstream effector mechanisms that regulated DNAPKcs. And that is the focus of our study.

4: Several expressions of control are not equally presented. I would suggest to repeat these data. Example: figure 2b, time point 0 of control and siCTDSP1. Figure 2b, time point 0 of control and CTDSP1. Figure 5, time point 0 of 0, 10 and 20uM drug treatment.

Re: I agree with the reviewers suggestion that there is a difference in protein quantity of topoI at 0 time points. However upon quantitation we have realized the visual difference is very minor and rate of degradation is statistically significant.

5: Immunofluorescence (IF) is not the appropriate method to quantify protein expression changes. All the protein expression of IF should be measured by flow cytometry, in order to understand both protein expression and cell population.

Re: We agree with the reviewer that IF may not be quantitative. However, recent developments in multiplexing largely depends on IF quantitation. We would also like to mention that we are not inferring the quantitative but visual estimation. TopoI is a nuclear protein and our effort to quantitatively analyze topoI-GFP by flow cytometry was not very successful.

**Reviewer #3:**

1. Instruments, chemicals and reagents used in experiments shall be clearly described with information manufacturers including name of manufacturers and location in the section of materials and methods. Some of them only stated in results and figure legends such as Leica SP5 confocal microscope and EGFP.
Re: Based on the suggestions of the reviewers we have revised the materials and methods to include the pertinent information on supplier and location of the manufacturers.

2. Line 182, cell density for each cell type shall be described clear.

Re: We have described the suggested cell density.

3. Replication numbers of each experiment shall be described by using proper statistical analysis to verify the differences among treatment conditions. Particularly the results of immunoblottings shall contain quantification data (E.g. Measurement of intensity) to compare the differences between cell types, treatments and experimental conditions with statistical analysis as well.

Re: As mentioned before we have included the suggested changes including immunoblot quantification.

4. Methodology of statistics for this manuscript is completely missed. Statistical analysis for both in vitro and in vivo studies shall be clearly described in an independent paragraph of materials and methods. Authors claimed many significant differences and effects among experiments but gave no statistical results for verification.

Each blots were quantified using Image J software (NIH, Bethesda, MD, USA). The statistical analyses were performed by using the JMP 14 statistical software package (SAS institute, Cary, NC, USA).  The Student t-test, the chi-square test, Fisher’s exact test and ANOVA one-way test were used where appropriate.

5. This manuscript was not written in a proper academic English and organization. For example, the aims of study was not clearly described in introduction, and too many active voices and questing among writing. Professional English editing service therefore is required to make this manuscript more readable.

Re: We have taken great care to edit and revise the manuscript

Sincerely,

Koji Ando, Department of Surgery and Science,
Kyushu University, 3-1-1 Maidashi, Higashi-ku,
Fukuoka 812-8582, Fukuoka, Japan

Tel: +81-92-642-5466

Fax: +81-92-642-5482

E-mail: k-ando@surg2.med.kyushu-u.ac.jp
