# Supplementary material for: CTDSP1 inhibitor rabeprazole regulates DNA-PKcs dependent topoisomerase I degradation and irinotecan drug resistance in colorectal cancer
Source: PLoS One. 2020 Aug 7;15(8):e0228002. doi: 10.1371/journal.pone.0228002 (PMC7413750; doi:10.1371/journal.pone.0228002)
Supplement: Attachment — Submitted filename: Response to Reviewers.docx [file pone.0228002.s005.docx]

May 31 2020

Joerg Heber

Editor-in-Chief

*PLOS ONE*

Dear Dr. Yu-Jia Chang,

Thank you for inviting us to submit a revised draft of our manuscript entitled, “CTDSP1 inhibitor rabeprazole regulates DNA-PKcs dependent topoisomerase I degradation and irinotecan drug resistance in colorectal cancer.” to PLOS ONE. We also appreciate the time and effort you and each of the reviewers have dedicated to providing insightful feedback on ways to strengthen our paper. Thus, it is with great pleasure that we resubmit our article for further consideration. We have incorporated changes that reflect the detailed suggestions you have graciously provided. We also hope that our edits and the responses we provide below satisfactorily address all the issues and concerns you and the reviewers have noted.

To facilitate your review of our revisions, the following is a point-by-point response to the questions and comments delivered in your letter.

**Reviewer #2**: This is the second-round review of this manuscript. Unfortunately the authors failed to appropriately response the comment from the first-round review.
4. The experimental procedure to generate TopoI-EGFP fusion by CRISPR/Cas9 system is not clearly presented. Please provide the sequence of sgRNA used in this study and sequencing result for the gene fusion.

- We agree with the reviewer that more detailed information is required to demonstrate the robust nature of our gene editing technology. We would like to emphasize the genome editing was performed in collaboration with Dr. Keith Jhoung’s Lab at Mass General Hospital and Harvard Medical School, Boston. His lab has published the details of methods and sequences of the vectors used (Kleinstiver BP et al 2015). We have included the sgRNA sequences. The sgRNA is MMW134. For the fusion gene sequence, we have already published in Ando et al., Oncogene 2017. Thus we will not be able to republish the data requested by the reviewer.

A well hTOP1 gene edit efficiency analysis and protein expression analysis should be performed in this manuscript. A previous study may be taken as an example. HDAC1,2 Knock-Out and HDACi Induced Cell Apoptosis in Imatinib-Resistant K562 Cells. Int J Mol Sci. 2019 May 8;20(9). pii: E2271. doi: 10.3390/ijms20092271.

- The suggested citation is an excellent work and a very good example to follow. However, in our case, the details of our genome editing technology were published by our collaborator. In the revised submission, we have included that reference. also published the technology (Ando et al. 2017). In our publication, we have provided the data on gene editing efficiency and protein expression data in the genome edited cells. Thus we will not be able to republish the data requested by the reviewer.

With the second review, some critical issues raised in the revised manuscript and the comments need to be appropriately responded.
1: The photos of clonogenic assay in figure 1C, 3C should be attached in supplementary data.

- As suggested, we have moved Fig. 1C to the supplementary data, but Fig. 3C was a cell viability assay.

2: It is very confusing of the following description “A comparison of two CRC cell lines, HCT116 and HT29, indicated higher expression of CTDSP1 in HCT116. A comparison of two CRC cell lines, HCT116 and HT29, indicated higher expression of 3 CTDSP1 in HCT116. We also observed minimal proteasomal degradation and HCT116 and HCT116 cells are significantly more sensitive to SN-38.” Page 9, line 2-4.

- As suggested, we have edited this section in the revised manuscript.

3: The significant TopoI protein expression drop is occurred after 2.5 μM SN-38 treatment in HCT116 cell (figure 1B). However, the bar figure doesn’t reflect this protein change. The results from blot and bar figure are not consistent.

-The revised Fig 1B reflects the similar topoI protein level and is consistent with the bar figure.

4: The protein expression blot of topoI-GFP protein in figure 2C and 5B should be revealed in figures.

- We appreciate the reviewer’s suggestions, but topoI-GFP immunoblotting will not add any additional information on the rate of topoI degradation. The purpose of including GFP-topoI fluorescence data is to support the immunoblotting data of Fig.2B and Fig. 5A at the single cell level and thus, immunoblotting of topoI-GFP to support Fig. 5C and 5B will be redundant.

5: The CTDSP1 expression in figure 1B, 2B, 3B, 4B, and 5A should also be revealed.

- This suggestion stems from the possibility of either inaccurate loading or potential degradation of CTDSP1 in response to SN-38. We have included β-actin immunoblot data in each Fig. and CTDSP1 does not degrade in response to SN-38. Thus, CTDSP1 immunoblotting will not provide any additional information to support our findings.

6: In figure 3B, a very significant topoI increase was observed in cells overexpressing CTDSP1, the description in result section is misleading.

- The increase on immunoblot appears to be high, but quantitative analysis indicated the increase is not significant.

7: A western blot result of pDNAPKcs expression in figure 4C should be revealed in the same figure.

- Fig. 4B and 4C are complementary: they both demonstrate the pDNA-PKcs in siCTDSP1 cells and they are in the same Fig.

8: The orders of the cell input in blot should consistent in this manuscript. For example: figure 2A is different from other figures (control, si or overexpression).

- As suggested, we have corrected the discrepancies.

9: IRB approval number is nor provided in the revised manuscript.

- We have included the IRB number in our revised manuscript.
